# SARS-CoV-2 cell tropism and multiorgan infection

**DOI:** 10.1038/s41421-021-00249-2

**Published:** 2021-03-23

**Authors:** Jia Liu, Yufeng Li, Qian Liu, Qun Yao, Xi Wang, Huanyu Zhang, Rong Chen, Liang Ren, Juan Min, Fei Deng, Bing Yan, Liang Liu, Zhihong Hu, Manli Wang, Yiwu Zhou

**Affiliations:** 1 Department of Forensic Medicine, Tongji Medical College of Huazhong University of Science and Technology, Wuhan, Hubei 430010 China; 2 State Key Laboratory of Virology, Wuhan Institute of Virology, Center for Biosafety Mega-Science, Chinese Academy of Sciences, Wuhan, Hubei 430071 China; 3 Jinyin-tan Hospital, Wuhan, Hubei 430023 China

**Keywords:** Cellular imaging, Cell biology

Dear Editor,

To date, the number of confirmed coronavirus disease 2019 (COVID-19) cases has surpassed 100 million, with deaths exceeding 2 million, yet the mechanism by which severe acute respiratory syndrome coronavirus (SARS-CoV)-2 attacks the body remains unclear. Although SARS-CoV-2 is known to primarily target the lung, it is also believed to cause multi-organ dysfunction and comprehensive studies on SARS-CoV-2 cell tropism in humans are lacking. SARS-CoV-2 exploits the host angiotensin-converting enzyme 2 (ACE2) as its receptor for cell entry^1^, but the correlation between SARS-CoV-2 organ/cell tropism and ACE2 distribution is unclear. Here, we studied these issues via a systemic analysis of postmortem specimens from a 66-year-old female COVID-19 patient who had rapidly developed multiorgan failure. The patient died in the hospital on Day 13 of admission (Day 16 of illness) and her autopsy was performed at 8 h after death.

To elucidate SARS-CoV-2 tissue tropism, we used immunohistochemical and immunofluorescence staining. Results showed that viral antigens (spike proteins) were highly expressed in pneumocytes and hyperplastic cells around the bronchioles (Supplementary Fig. S1a–c); mucosal epithelia, submucosal glands, and gland ducts of the trachea (Supplementary Fig. S1d–f); mucosal epithelia and glands of the small intestine (Supplementary Fig. S1g, i); distal tubules and collecting ducts of the kidneys (Supplementary Fig. S1j–l); islets of Langerhans, glands, and intra-islet ducts of the pancreas (Supplementary Fig. S1m, n); and vascular tissues of the brain (Supplementary Fig. S1o, p) and heart (Supplementary Fig. S1q, r). In contrast, few viral antigens were present in the large intestine (Supplementary Fig. S1h) and renal proximal tubules (Supplementary Fig. S1j), and none in the liver (Supplementary Fig. S1s, t). Collectively, these data demonstrate direct multiorgan invasion by, or exposure to, SARS-CoV-2.

To determine the relationship between SARS-CoV-2 organotropism and receptor ACE2 distribution, we performed a co-localization analysis. Co-expression of ACE2 and viral antigen was observed in the lung, trachea, small intestine, kidney, pancreas and heart (Fig. 1a; Supplementary Fig. S2a-i, ii). In the brain, ACE2-expressing cells were detected, but they did not appear to be viral antigen-positive (Supplementary Fig. S2a-iii). In contrast, ACE2 was not expressed in the liver (Supplementary Fig. S2a-iv). These findings suggest that SARS-CoV-2 largely exploits ACE2 as receptor for cell entry in multiple organs.

**Fig. 1:**
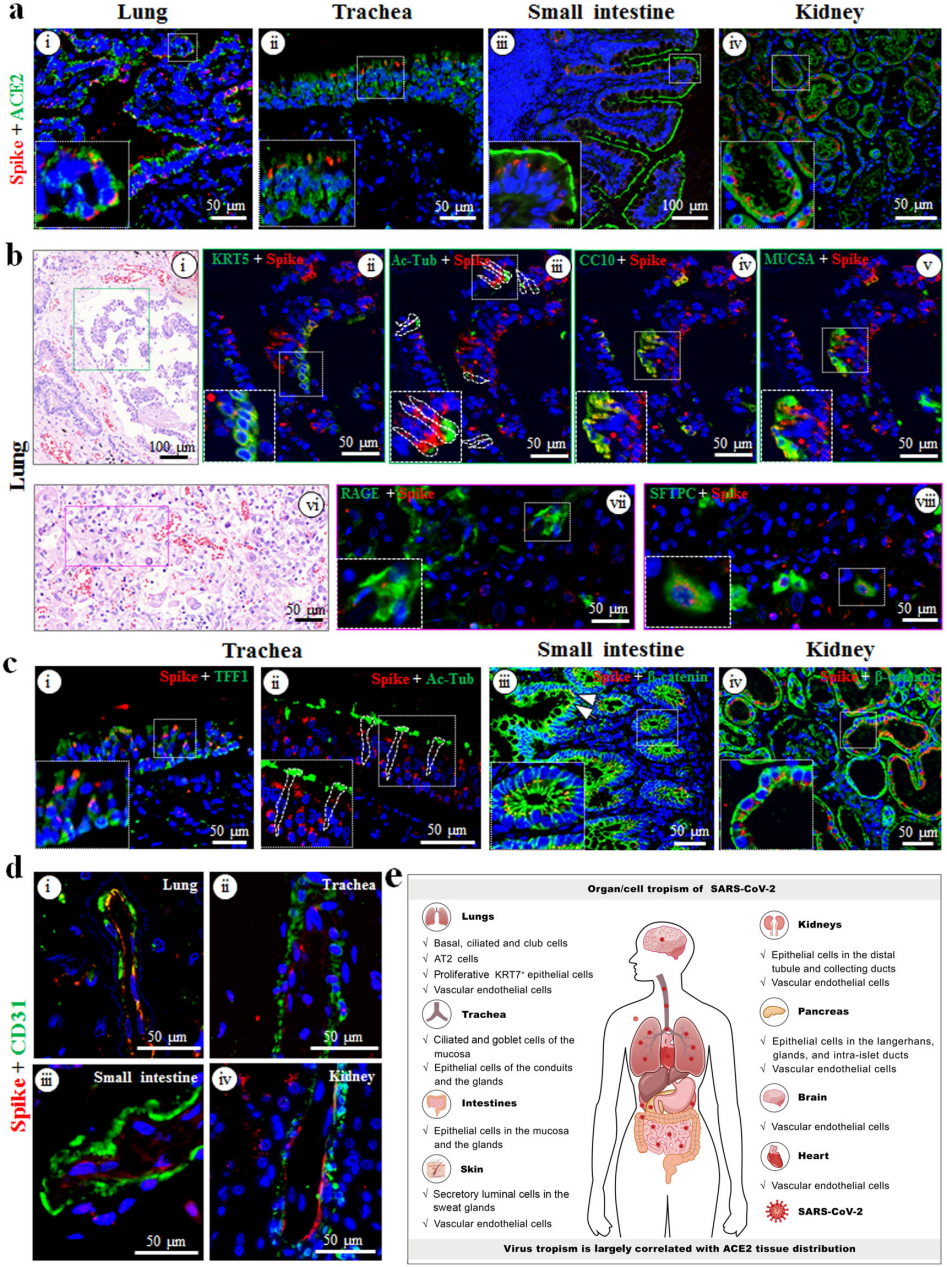
Detection of multiorgan infection and cell tropism of SARS-CoV-2 in postmortem samples. **a** Co-localization analysis of SARS-CoV-2 (spike proteins, red) and ACE2 (green) in the lung (i), trachea (ii), small intestine (iii), and kidney (iv) via dual-immunofluorescent staining. **b** Characterization of SARS-CoV-2-positive cell tropism in the lung. Co-localization of SARS-CoV-2 (spike proteins, red) with cell type-specific markers (green) was determined via multiplex-immunofluorescence staining. KRT5 (ii), Ac-Tub (iii), CC10 (iv), MUC5A (v), RAGE (vii), and SFTPC (viii) were used for basal, ciliated, club, goblet, AT1, and AT2 cells, respectively. The corresponding hematoxylin and eosin staining figures were shown in (i) and (vi), respectively. **c** Characterization of SARS-CoV-2 positive cell types in the trachea (i and ii), small intestine (iii), and kidney (iv). TFF1 was used for goblet cells (i), and Ac-Tub for ciliated cells (ii) in the trachea; β-catenin was used for epithelial cells in small intestine (iii) and kidney (iv). White arrows (iii) indicate the mucosa of the small intestine. **d** Co-localization analysis of SARS-CoV-2 (spike proteins, red) with CD31^+^ vascular endothelial cells (green) in the lung (i), trachea (ii), small intestine (iii), and kidney (iv). **e** Schematic of SARS-CoV-2 multiorgan infection and cell tropism in the human body.

To elucidate the details of SARS-CoV-2 cell tropism in different tissues, we analyzed the co-localization of viral antigens with various cell lineage markers by using multiplex immunofluorescence assay. In the lung, viral antigens were detected in major epithelial cell types of the bronchi/bronchioles including keratin 5-positive (KRT5^+^) basal, acetylated α-tubulin antibody-positive (Ac-Tub^+^) ciliated, and club cell 10-positive (CC10^+^) club cells (Fig. 1b-i–iv). Although we found that some goblet marker mucin 5A (MUC5A)-positive cells were infected (Fig. 1b-v), these cells also co-expressed CC10 (Fig. 1b-iv), suggesting they might be transdifferentiated/differentiated from club cells. The virus also infected a few receptor for advanced glycation end products–positive (RAGE^+^) alveolar type (AT)1 cells as well as surfactant protein C-positive (SFTPC^+^) AT2 cells (Fig. 1b-vi–viii). Some viral positive cells were found to co-express both AT1 and AT2 cell markers, but were morphologically distinct from the flat mature AT1 cells (Supplementary Fig. S2b-i, ii), suggesting the infected AT1 cells might be transdifferentiated/differentiated from AT2 cells. Interestingly, viral antigen signals were detected at high levels in many proliferative KRT7^+^ epithelial cells (Supplementary Fig. S2c-i) and in a few Ki67^+^ proliferative cells (Supplementary Fig. S2c-ii). Collectively, our results suggest that SARS-CoV-2 mainly infected basal, ciliated, club, AT2, and proliferative KRT7^+^ epithelial cells in the lung.

In the trachea, epithelial marker KRT7 clearly revealed viral infection in epithelial cells of the mucosa (Supplementary Fig. S3a-i), conduits, and glands (Supplementary Fig. S3a-ii). Among mucosal epithelial cells, viral antigens were found in trefoil factor 1-positive (TFF1^+^) goblet (Fig. 1c-i) and Ac-Tub^+^ ciliated cells (Fig. 1c-ii), but few were found in KRT5^+^ basal (Supplementary Fig. S3a-iii) and CC10^+^ club (Supplementary Fig. S3a-iv) cells. In the small intestines, viral antigens were abundant in mucosal (white arrows) and glandular (white box) β-catenin^+^ epithelial cells (Fig. 1c-iii). In the kidneys, β-catenin^+^-distal tubule (Fig. 1c-iv) and collecting duct (Supplementary Fig. S3b-i) epithelial cells were highly sensitive to SARS-CoV-2 infection. CD31^+^ glomerular endothelial cells also showed positive signals for SARS-CoV-2 (Supplementary Fig. S3b-ii), but viral antigens were not detected in the proximal tubules (Supplementary Fig. S3b-iii).

The virus infected the CD31^+^ vascular endothelial cells in the lung, trachea, small intestine, and kidney (Fig. 1d) as well as the pancreas, heart and brain (Supplementary Fig. S4a). As expected, ACE2 was observed in the vascular endothelial cells of different tissues (Supplementary Fig. S4b). Taken together, these results confirm that SARS-CoV-2 preferentially targets vascular endothelial cells in multiple organs.

It is known that the respiratory system is a major target of SARS-CoV-2. Recent studies showed that SARS-CoV-2 targets ciliated and AT2 cells in airway and alveolar regions^2^, and consistently epithelium ciliated cells and AT2 in lung are the major cell types that co-express ACE2 and co-receptor transmembrane protease seine 2 (TMPRSS2)^3^. Our study identified viral infection in basal-, ciliated-, club-, and AT2 cells in the lungs (Fig. 1b) and in epithelial goblet and ciliated cells of the trachea (Fig. 1c-i, ii). Proliferative hyperplastic cells in the lungs were among the major target cells (Supplementary Fig. S2c), which is in line with a recent report that identified newly proliferated cells in the respiratory epithelia of COVID-19 patients^4^. It is likely that those cells were proliferated upon viral infection, or they were generated to repair lung damage but were hijacked for virus amplification.

Expressions of ACE2 and TMPRSS2 in the lungs and trachea were further analyzed. In the lung, ACE2 and TMPRSS2 were easily detected in some ciliated-, club- and goblet cells, and many of them were viral-positive **(**Supplementary Fig. S5b–d). However, ACE2 and TMPRSS2 were rarely detected in the basal cells where virus infection virtually occurred (Supplementary Fig. S5a), suggesting the entry factors by which the virus exploits in basal cells need further investigation. Co-expression of ACE2 and TMPRSS2 was identified in some AT2 cells and consistently, viral infection was detected (Supplementary Fig. S5f). By contrast, ACE2 and TMPRSS2 were rarely detected in AT1 cells (Supplementary Fig. S5e), which may explain why SARS-CoV-2 was hardly detected in AT1 cells. The proliferative KRT7^+^ cells co-expressed high levels of ACE2 and TMRPSS2, supporting the finding that these cells were permissive for virus infection (Supplementary Figs. S5g and S2c-i). In trachea, ACE2 and TMPRSS2 could be easily detected in ciliated-, and goblet cells, and consistently, many of them were viral positive (Supplementary Fig. S6a, b). Therefore, except for the basal cells in the lung, our results generally supported the correlation between ACE2/TMPRSS2 distribution and SARS-CoV-2 cell tropism in the lung and trachea. The discrepancies between our results and other studies^2,3^, may be due to different methodologies and different human samples used. For example, ACE2 expression appeared to be induced by interferon or dysregulated in patients with pre-existing pulmonary diseases^2,3^.

Accumulating evidence suggests that the intestines might be a target organ^5^ and gastrointestinal symptoms have been reported in some COVID-19 patients. Here, high ACE2 expression levels and an abundance of viral antigens were consistently detected in enterocytes of the small intestine (Fig. 1a-iii), which concurs with a report that ACE2 expression was high in the microvilli of intestines^6^. Our findings are also supported by studies showing successful isolation of SARS-CoV-2 from the stool samples of patients with severe COVID-19^7^. Collectively, the evidence implies that the gastrointestinal system might serve as a SARS-CoV-2 transmission route and amplification factory.

Acute kidney injury has been reported in up to ~25% of critically-ill COVID-19 patients^8^, and SARS-CoV-2 has been isolated from the urine samples of some patients^9^. We observed high ACE2 expression in the proximal, distal tubules, and collecting ducts (Fig. 1a-iv); however, viral antigens were only identified in the distal tubules and collecting ducts, not in the proximal tubules (Fig. 1c-iv; Supplementary Figs. S1j–l and S3b-iii). This distribution pattern is similar to that of SARS-CoV^10^, but is different from some studies showing SARS-CoV-2-infected proximal tubules^11^. This suggests that in addition to ACE2, other host factors, such as co-receptors, immune response and viral transmission, may also play critical roles for virus infection in these tissues. It remains unknown whether the viral distribution we observed is case specific. Nevertheless, our data and those in other reports suggest that acute kidney injury is a pathological consequence of direct viral infection and that urine is a possible SARS-CoV-2 transmission route.

Vascular endothelial injury, endotheliitis, and coagulation dysfunction have been reported in some COVID-19 patients; such conditions are likely associated with disease severity^12^. Actually, direct SARS-CoV-2 infection of lung microvascular endothelial cells in human and cultured blood vessel organoids has been reported^12–14^. We detected SARS-CoV-2 infection in the CD31^+^ vascular endothelial cells of multiple organs (Fig. 1d; Supplementary Fig. S4a). ACE2 has been reported in arterial and venous endothelial cells;^14^ likewise, we detected ACE2 expression in the vascular endothelial cells of multiple organs (Supplementary Fig. S4b). The fact that SARS-CoV-2 infection in the vascular endothelial cells of various organs induces both direct and indirect damage (e.g., coagulation dysfunction) suggests that blood vessels are important target tissues that might represent a dissemination route within the human body.

In conclusion, our results identified SARS-CoV-2 cell tropism in multiple organs (Fig. 1e), indicating that SARS-CoV-2 infects not only the respiratory system (e.g., lungs and trachea) but also the kidneys, small intestines, pancreas, blood vessels, and other tissues. Recently, we disclosed that SARS-CoV-2 also targeted sweat glands and vascular endothelial cells in the skin^15^. These findings suggest that direct viral infection could, at least partially, contribute to multiorgan injury. Our results also proved a possible correlation between SARS-CoV-2 organotropism and ACE2 distribution, providing supporting evidence for the multiorgan infection of the virus. While our study was limited by its size, its sheds new light into the mechanism of viral infection/transmission and provides valuable information for COVID-19 control.

## Supplementary information

Supplementary Information
