# Supplementary material for: SARS-CoV-2 cell tropism and multiorgan infection
Source: Cell Discov. 2021 Mar 23;7:17. doi: 10.1038/s41421-021-00249-2 (PMC7987126; doi:10.1038/s41421-021-00249-2)
Supplement: Supplementary file 1 — Supplementary Information [file 41421_2021_249_MOESM1_ESM.pdf]

SARS-CoV-2 cell tropism and multiorgan infection

Jia Liu[1,2†], Yufeng Li[2,†], Qian Liu[1,†], Qun Yao[3,†], Xi Wang[2], Huanyu Zhang[2], Rong Chen[3], Liang Ren[1], Juan Min[2], Fei Deng[2], Bing Yan[2], Liang Liu[1], Zhihong Hu[2,*], Manli Wang[2,*], Yiwu Zhou[1,*]

Supplementary Appendix: Table of Contents

Page

Materials and Methods

Histochemical staining..2

Imunohistochemical (IHC) staining..2

Immunofluorescence (IFA) staining..2

Multiplex Immunofluorescence Assay..3

Supplementary Figures

Fig. S1 SARS-CoV-2 detection in different organs. 4

Fig. S2 Co-localization analyses of SARS-CoV-2 spike protein with receptor ACE2 or different cell markers in multiple organs..5

Fig. S3 Characterization of SARS-CoV-2 positive cell types in the autopsied trachea and kidney. .6

Fig. S4 SARS-CoV-2 infection of vascular endothelial cells in different organs and its colocalization with receptor ACE2..7

Fig. S5 Co-localization of SARS-CoV-2 with different cell markers and its receptor/co-receptor in the lung..8

Fig. S6 Co-localization of SARS-CoV-2 with different cell markers and its receptor/co-receptor in the trachea. 9

Supplementary Tables

Table S1. Clinical laboratory data..10

Table S2. Information of antibodies used for IHC and IFA 11

Materials and Methods

Histochemical staining

Lung tissue were collected and fixed in 10% neutral buffered formalin. Tissues were routinely processed for paraffin embedding and sectioning. Then serial sections (3 μm-thick) of formalin fixed paraffin-embedded (FFPE) tissue blocks were obtained and stained with hematoxylin and eosin (H&E).

Imunohistochemical (IHC) staining

Immunohistochemical staining was performed on the sections for each organ samples. Briefly, after deparaffinization, heat-mediated antigen retrieval in citrate buffer (pH: 6.0) (Sigma-Aldrich) by microwave was applied on FFPE sections. After cooling and rinsing with distilled water, quenching of endogenous peroxidase was performed with 0.3% hydrogen peroxide for 15 min at room temperature. The section was washed in water, and blocked with phosphate-buffered saline (PBS), pH7.4 containing 5% bull serum albumin (BSA) for overnight at 4 °C. Then the primary antibodies against SARS-CoV-2 spike proteins were diluted in PBS containing 1% BSA at corresponding concentration (Table S2) and incubated over night at 4 °C. After washing with PBS for three times, the sections were incubated with horseradish peroxidase (HRP)-conjugated goat anti-rabbit/mouse IgG antibody (1:500; Proteintech, China) as second antibody. For chromogenic DAB staining, slides were incubated with peroxidase complex according to the manufacturer's instructions of RBD staining kit (Servicebio), followed by counterstaining with hematoxylin. The diluent without primary antibodies was used as negative control. Images were captured using a digital camera under a light microscope (Thermo, Chriscan-SF).

Immunofluorescence (IFA) staining

After deparaffinization, the FFPE sections were treated with 0.1% Sudan Black B (Sigma-Aldrich) for 15 min at room temperature to reduce autofluorescence. Then the

tissues were heated in citrate buffer (pH6.0) (Sigma-Aldrich) by microwave for antigen retrieval. After rinses with PBS, sections were blocked overnight with PBS containing 5% BSA at 4 °C. Sections were then incubated with primary antibodies at corresponding dilution (Table S2) overnight at 4 °C. After washing with PBS for three times, sections were incubated with secondary Alexa Fluor 488/555/647-conjugated goat anti-rabbit or mouse IgG (Abcam) for 2 h at room temperature. Sections were stained with DAPI (Biyuntian) and cover-slipped using Antifade Mounting Medium (Servicebio). Images were captured using a digital camera under a light microscope (Thermo, Chriscan-SF).

Multiplex Immunofluorescence Assay

Three μm paraffin sections were deparaffinized in xylene and rehydrated in a series of graded alcohols. Antigen retrievals were performed in citrate buffer (pH6.0) using microwave for 20 min at 98 °C followed by free cooling at room temperature. Multiplex fluorescence labeling was performed by TSA-dendron-fluorophores (opal 7-color Manual IHC kit, Perkinelmer, NEL811001kt). Briefly, after antigen retrieval, endogenous peroxidase of the section was quenched in 3% H_2O_2 for 20 min. Then the section were washed with TBST for 3 min, followed by incubating with blocking buffer for 10 min at room temperature. Primary antibody was incubated for 1 hour at room temperature, followed by detecting with the HRP-conjugated secondary antibody and TSA-dendron-fluorophores. Before detection for another antigen, the conjugated primary and secondary antibodies were removed from the section by heating in retrieval buffer using microwave as mentioned above. All the antigens, including (SARS-CoV-2 Spike protein, KRT5, Ac-Tub, CC10, MUC5A, KRT7, SFTPC, RAGE, Ki67, TFF1, β-Catenin, CD31, ACE2, and TMRPSS2) (Table S2) were labeled by distinct fluorophores sequentially, then the sections were stained with DAPI (Biyuntian) and cover-slipped using Antifade Mounting Medium (Servicebio) and imaging using Perkinelmer VECTRA3 automatic quantitative analysis system.

Supplementary Figures

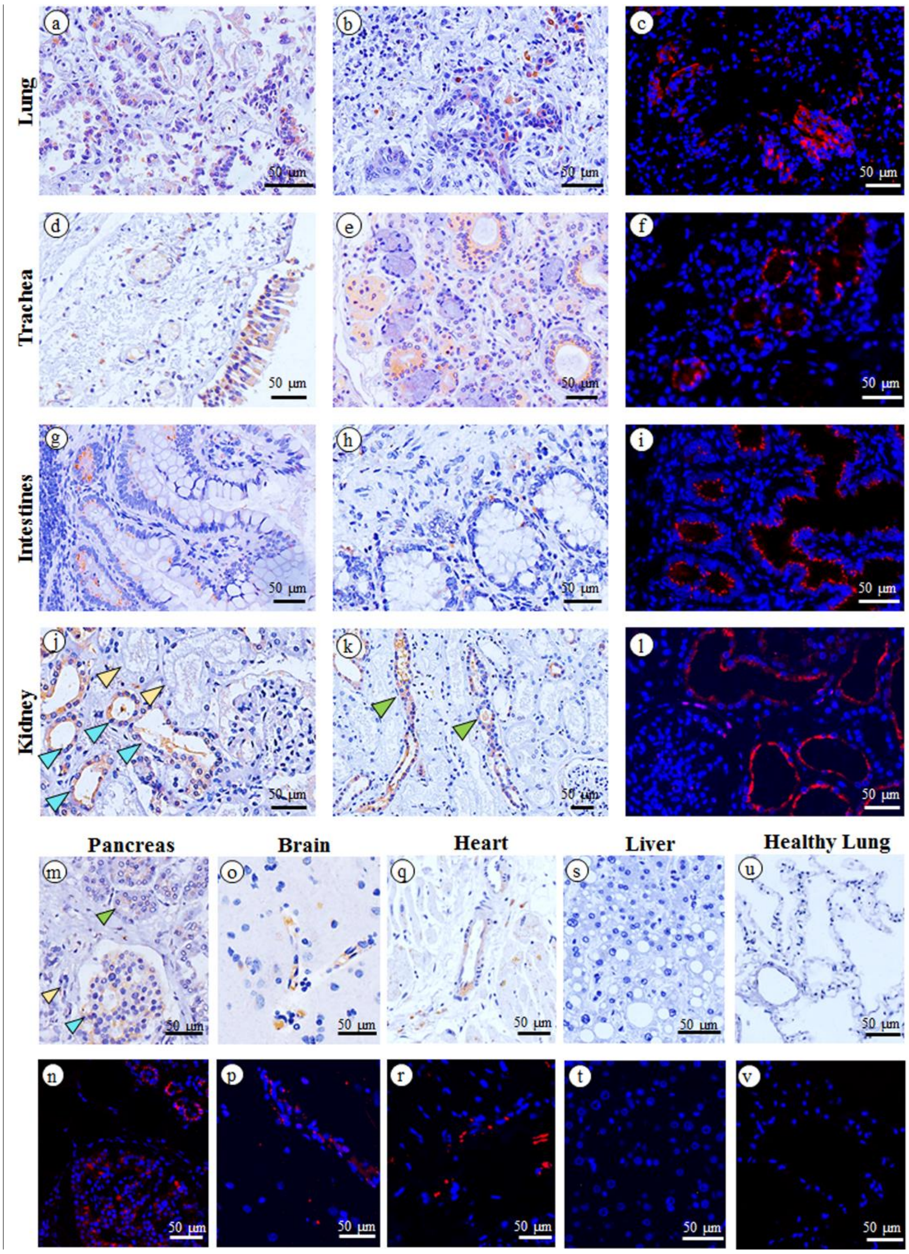

Fig. S1 SARS-CoV-2 detection in different organs.

Immunohistochemical and immunofluenrence analyses of SARS-CoV-2 infection in multiple organs. The distribution of viral antigen in various tissue, including lung (a-c), trachea (d-f), gut (g-i), kidney (j-l), pancreas (m-n), brain (o-p), heart (q-r), and liver (s-t) was performed using antibody against SARS-CoV-2 spike protein. A healthy lung tissue was used as negative control (u-v). The yellow, blue and green arrows in (j and k) indicated the proximal, distal tubules, and collecting ducts, respectively. The blue, green and yellow arrows in (m) indicated the islets of Langerhans, glands (green arrowhead), and intra-islet ducts, respectively.

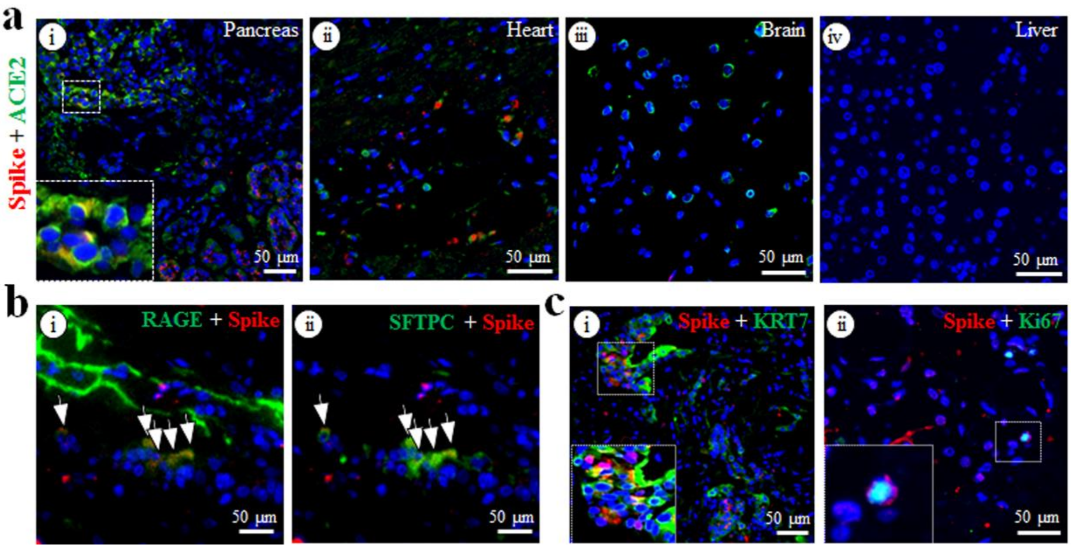

Fig. S2 Co-localization analyses of SARS-CoV-2 spike protein with receptor ACE2 or different cell markers in multiple organs.

a Co-localization analysis of SARS-CoV-2 (spike protein, red) with ACE2 (green) in the pancreas (i), heart (ii), brain (iii), and liver (iv) was performed by dual-immunofluorescent staining. **b-c** Co-localization of SARS-CoV-2 (red) with cell type–specific markers (green) was determined via dual/multiple-immunofluorescencent staining. RAGE (b-i) and SFTPC (b-ii) were used for AT1 and AT2 cells, respectively; KRT7 (c-i) and Ki67 (c-ii) were used for proliferative cells. The inserted small image is the enlarged image of the white box in the large image in a-i, and c-i-ii.

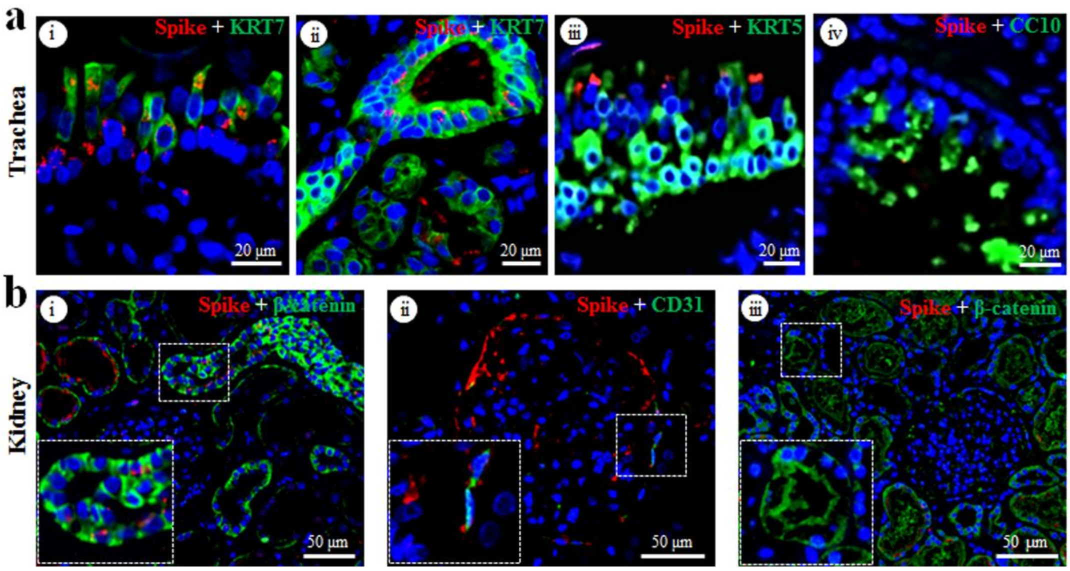

Fig. S3 Characterization of SARS-CoV-2 positive cell types in the autopsied trachea and kidney.

a Co-localization of SARS-CoV-2 (spike proteins, red) with cell-type-specific markers (green) in the trachea determined by dual-immunofluorescent staining, including KRT7 for epithelial cells in mucosal epithelia (i), gland ducts and submucosal glands (ii); KRT5 for basal cells (iii), and CC10 for club cells (iv). **b** Co-localization analysis of SARS-CoV-2 with marker (β-catenin) of the renal collecting tubule (i) and proximal tubule (iii), and with marker of endothelial cells (CD31) in the glomerulus of the kidney (ii). The inserted small image in the left bottom was the enlarged image of the white box in the large image in b-i-iii.

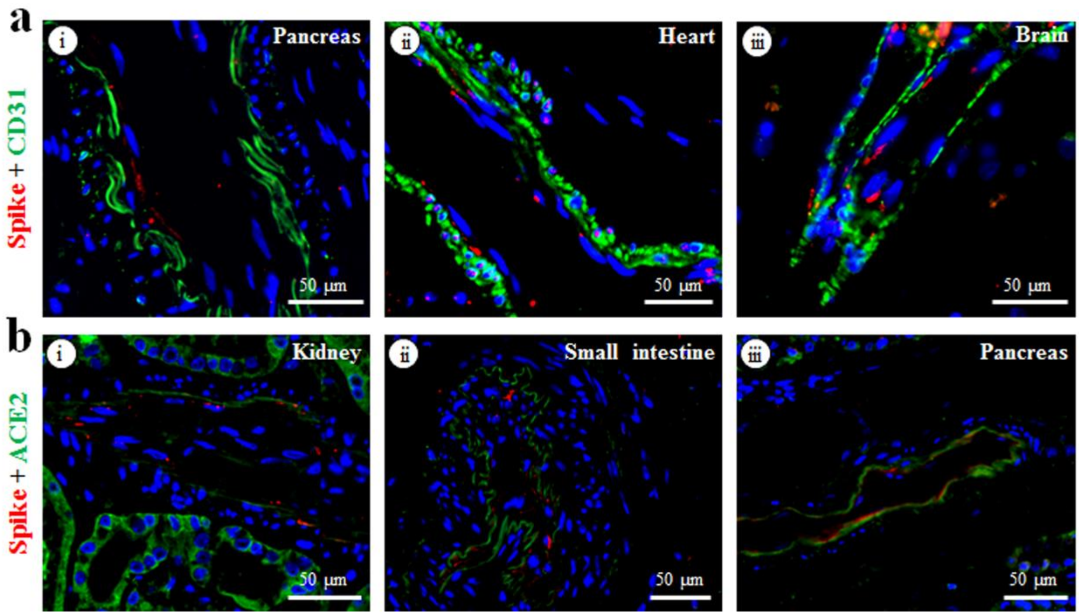

Fig. S4 SARS-CoV-2 infection of vascular endothelial cells in different organs and its colocalization with receptor ACE2.

a Co-localization analysis of SARS-CoV-2 (spike proteins, red) with the marker (CD31, green) for vascular endothelial cells in pancreas (i), heart (ii), and brain (iii) by dual-immunofluorescent staining. **b** Co-localization analysis of SARS-CoV-2 (spike proteins, red) with ACE2 (green) in the kidney (i), small intestine (ii), and pancreas (iii) by dual-immunofluorescent staining.

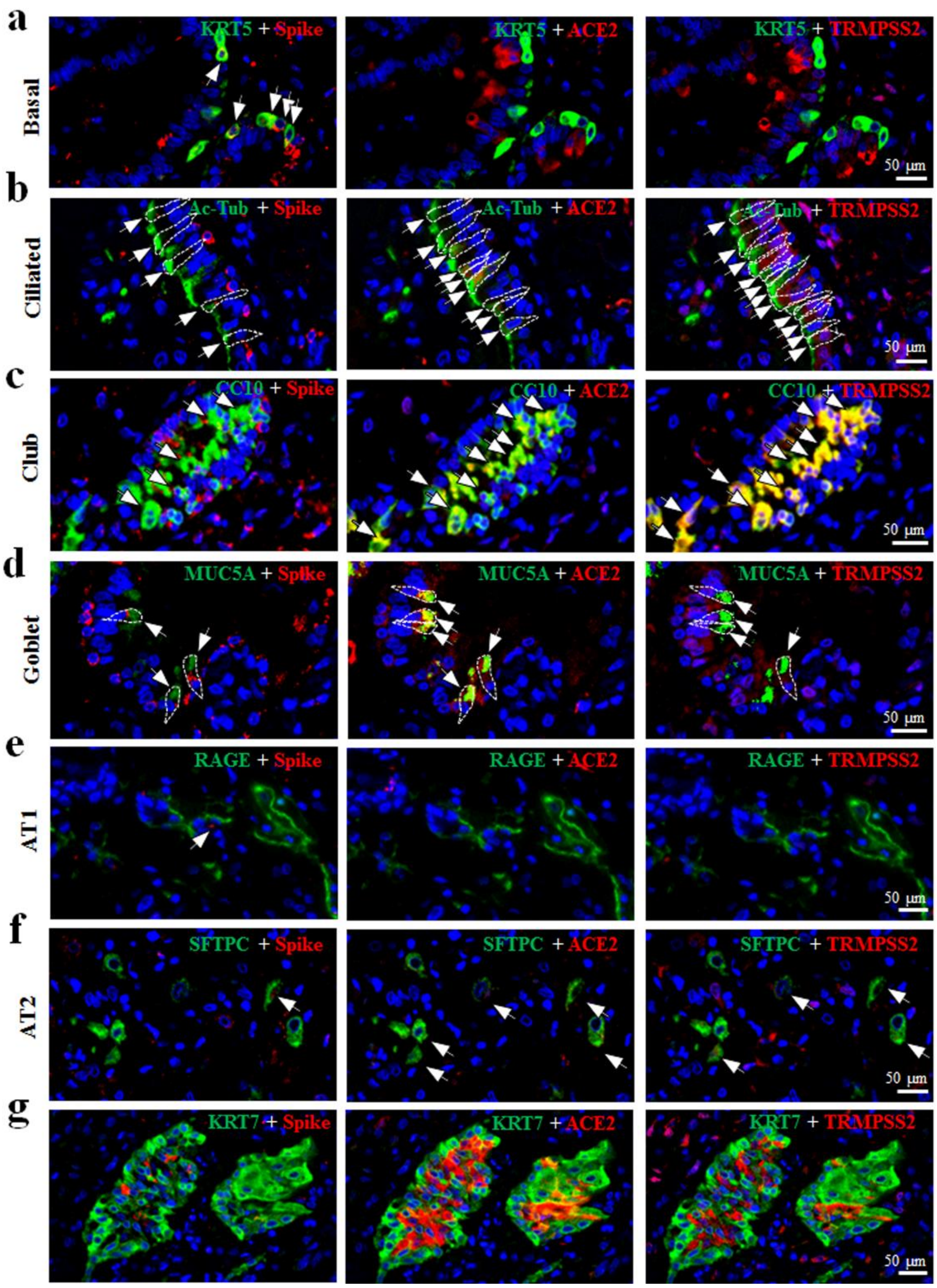

Fig. S5 Co-localization of SARS-CoV-2 with different cell markers and its receptor/co-receptor in the lung.

Co-localization of SARS-CoV-2 (spike proteins, red) with cell type–specific markers/ACE2/TRMPSS2 (green) was determined via multiplex immunofluorescence staining. KRT5 (a), Ac-Tub (b), CC10 (c), MUC5A (d), RAGE (e), and SFTPC (f) were used for basal, club, ciliated, goblet, AT1, and AT2 cells, respectively. The white dashed line indicated the cell boundary in b and d. Arrows indicated co-localization of the two indicated markers in each figure.

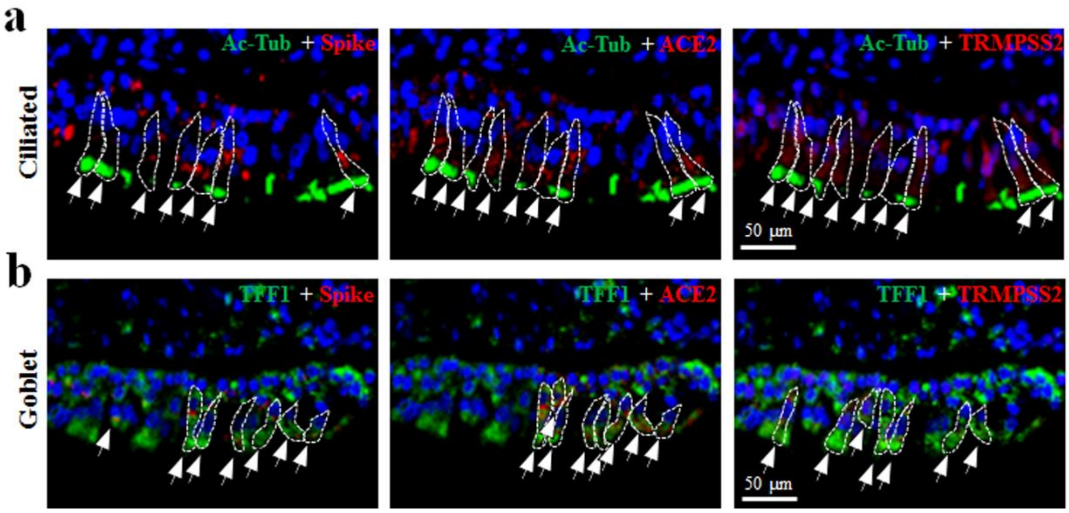

Fig. S6 Co-localization of SARS-CoV-2 with different cell markers and its receptor/co-receptor in the trachea.

Co-localization of SARS-CoV-2 (spike proteins, red) with cell type–specific markers/ACE2/TRMPSS2 (green) was determined via multiplex-immunofluorescence staining. Ac-Tub (a), and TFF1 (b) were used for ciliated, and goblet cells, respectively. The white dashed line indicated the cell boundary. Arrows indicated co-localization of the two indicated markers in each figure.

Supplementary Tables

Table S1. Clinical laboratory data

Table S1. Clinical Laboratory Data				
Variable	Reference Range (adults)	4 days after onset (On admission)	9 days after onset	13 days after onset
Blood routine parameters				
Hemoglobin (g/L)	115-150	130	117	106↓
White cells (10^9/L)	3.5-9.5	22.39↑	37.5↑	18.99↑
Neutrophils (10^9/L)	1.8-6.3	19.68↑	35.94↑	17.28↑
Lymphocytes (10^9/L)	1.1-3.2	1.97	0.63↓	0.93↓
Monocytes (10^9/L)	0.1-0.6	0.66↑	0.87↑	0.55
Platelets (10^9/L)	125-350	289	84↓	63↓
Blood biochemical indexes				
Alanine aminotransferase (U/L)	7-40	88↑	46↑	114↑
Aspartate aminotransferase (U/L)	13-35	240↑	75↑	141↑
Albumin (g/L)	40-55	28.8↓	27.3↓	27↓
Direct bilirubin (umol/L)	0-8	3.9	8.3↑	17.4↑
Glutamyl transpeptidase (U/L)	7-45	41	97↑	84↑
Creatinine kinase (U/L)	40-200	2720↑	600↑	1673↑
Lactate dehydrogenase (U/L)	120-250	1361↑	1222↑	2150↑
Troponin (mm/h)	0-28	7449.2↑	119.9↑	210.6↑
Myohemoglobin (mm/h)	0-146.9	＞1200.0↑	＞1200.0↑	＞1200.0↑
Urea nitrogen (mmol/L)	2.6-7.5	14.5↑	42.38↑	＞35.6↑
Creatinine (mmol/L)	41-73	104.5↑	269↑	270.5↑
Triglycerides (mmol/L)	0.51-1.7	1.6	4.65↑	9.62↑
Potassium (mmol/L)	3.5-5.3	4.3	6↑	5.6↑
Sodium (mmol/L)	137-147	148↑	160↑	168↑
Coagulation index				
Prothrombin time (s)	10.5-13.5	13	15.1↑	19.2↑
International normalized ratio	0.8-1.2	1.1	1.35↑	1.77↑
Fibrinogen (g/L)	2-4	6.3↑	1.9↓	1.5↓
D-dimer (ug/mL)	0-1.5	5.56↑	17.78↑	12.73↑
Fibrinogen degradation product (ug/mL)	0-5	15.1↑	56.95↑	36.46↑
Inflammatory biomarkers and cytokine				
C-reactive protein (mg/L)	0-5	229.6↑	28.9↑	5.7
Ferritin (ug/L)	4.63-204	-	＞2000.0↑	＞2000.0↑
IL-6 (pg/mL)	0-7	-	8.05↑	21.45↑

Table S2. Information of antibodies used for IHC and IFA

Primary antibodies	Company	Catalog	Host	Dilutions
Spike proteins	Made in-house	--	Mouse	1:300
KRT5	Abcam	ab64081	Rabbit	1:100
CC10	Santa Cruz	sc-365992	Mouse	1:200
MUC5A	Abcam	ab198294	Mouse	1:200
Ac-Tub	Cell signaling tech	#5335	Rabbit	1:500
SFTPC	Sigma	HPA010928	Rabbit	1:300
RAGE	Abcam	ab216329	Rabbit	1:500
Ki67	Servicebio	GB13030-2	Rabbit	1:400
CD31	Boster	A01513-3	Rabbit	1:200
TFF1	Proteintech	13681-1-AP	Rabbit	1:100
KRT7	Cell signaling tech	#4465	Rabbit	1:100
β-Catenin	Csignaling tech	#8480	Rabbit	1:100
ACE2	Abcam	ab15348	Rabbit	1:200
